# The Impact of Telemonitoring and Telehealth Coaching on General Nutrition Knowledge in Overweight and Obese Individuals: A Pilot Randomized Controlled Trial

**DOI:** 10.3390/medsci12040068

**Published:** 2024-11-22

**Authors:** Noura M. S. Eid, Ebtisam A. Al-Ofi, Sumia Enani, Rana H. Mosli, Raneem R. Saqr, Karimah M. Qutah, Sara M. S. Eid

**Affiliations:** 1 Department of Clinical Nutrition, Faculty of Applied Medical Sciences, King Abdulaziz University, P.O. Box 80215, Jeddah 21589, Saudi Arabia; aimuwseli@kau.edu.sa; 2 King Fahd Medical Research Center, King Abdulaziz University, Jeddah 21589, Saudi Arabia; ealofi@kau.edu.sa (E.A.A.-O.); senani@kau.edu.sa (S.E.); eidsar@gmail.com (S.M.S.E.); 3 Department of Physiology, Faculty of Medicine, King Abdulaziz University, Jeddah 21589, Saudi Arabia; 4 Department of Food and Nutrition, Faculty of Human Sciences and Design, King Abdulaziz University, Jeddah 21589, Saudi Arabia; 5 Department of Management Information System, Faculty of Economics and Administration, King Abdulaziz University, Jeddah 21589, Saudi Arabia; rsaqr@kau.edu.sa; 6 The Management of Digital Transformation and Innovation Systems in Organization Research Group, King Abdulaziz University, Jeddah 21589, Saudi Arabia; 7 Horizon Health Network, Fredericton, NB 506, Canada; kqutah@gmail.com

**Keywords:** general nutrition knowledge, obesity, telenutrition, telemonitoring, telehealth coaching, online nutrition education

## Abstract

(1) Background: General nutrition knowledge is a fundamental pillar of well-being and healthy lifestyles. This study aimed to measure the general nutrition knowledge questionnaire (GNKQ) scores of overweight and obese participants who joined a pilot randomized controlled trial (RCT) and the association between changes in GNKQ scores and changes in anthropometric measures. (2) Methods: A total of 30 and 25 participants had completed the trial at the 3- and 6-month visits, respectively. All participants enrolled in a randomized controlled trial (RCT) and received a hypocaloric-tailored diet and three online nutrition education sessions over 6 months. The participants were randomly divided into two groups: an intervention group supported with weekly telemonitoring and monthly telehealth coaching vs. a control group. The Arabic-validated GNKQ was used, covering four sections: dietary recommendations; food groups and nutrient sources; healthy food choices; and associations between the diet–disease relationship and weight. (3) Results: The findings show that both the intervention and control groups showed improvements in GNKQ scores over time, with the intervention group demonstrating significant increases in overall nutrition knowledge and specific areas, such as the diet–disease relationship and weight management, at 3 months. In addition, changes in GNKQ scores had a significant negative association with BMI and visceral fat percentage. The findings underline the benefits of supporting dietary weight loss interventions with telemonitoring and telehealth coaching, suggesting that an increase in nutrition knowledge may relate to lower body fat metrics. Nevertheless, the small sample size and high attrition rate of participants were the main limitations of this study, such that large populations are required to confirm the reliability of the obtained findings.

## 1. Introduction

Health and nutrition knowledge has been identified as a global goal for all populations to develop healthy food relationships [1]. Research has proven that nutrition knowledge plays a major role in following healthier eating habits [2,3]. The main concerns identified are difficulties in reading food labels and choosing the healthiest food product options due to a lack of knowledge [4]. Globally, nutrition programs and resources are available for implementation in schools, as seen in the UK, USA, India, and Singapore [5]. Nevertheless, learning resources and nutrition education programs to support schools in implementing nutrition education are lacking in Saudi Arabia. Moreover, teachers have shown great interest and positive attitudes toward delivering nutrition education to students [6]. However, several obstacles have been identified by Saudi schools, including a lack of awareness and training, cost coverage, and resistance [7]. In a cross-sectional study, Saudi women showed low scores in nutrition knowledge, particularly in portion size and MyPlate guidelines [8]. Obesity also remains a major health burden worldwide [9]. Sedentary lifestyles and poor eating habits have been identified to be associated with behavior toward food and lack of knowledge [10]. The World Health Organization (WHO) has predicted that by 2030, almost 30% of death cases worldwide will be associated with “lifestyle diseases”, where strategies that target population behaviors, attitudes, and knowledge are essential [11]. Ensuring that the population is fully aware of nutrition and health information is essential to improving eating habits and attitudes toward nutrition and health [12]. Meanwhile, eating habits refer to conscious, collective, and repetitive behaviors that lead people to select, consume, and use certain foods or diets in response to social and cultural influences [13]. This dynamic interplay between nutrition knowledge and eating habits profoundly influences various health aspects, especially weight. For instance, a well-informed understanding of nutrition often leads to wiser food choices, ultimately impacting overall diet quality and nutrient intake. These informed choices, in turn, have far-reaching implications for preventing chronic diseases resulting from obesity and overweight, such as diabetes, cardiovascular conditions, and certain cancers [14]. Several factors influence the general nutrition knowledge of a population, including sex, educational background, obesity, and lifestyle [15]. According to research, nutrition knowledge sources vary, including medical sources and internet content, family members or friends, and TV, which may be misleading [16]. According to a previous study on general nutrition knowledge in the Austrian population, 41.4% of the general population misclassified sugar as the most calorific nutrient, while only 29% of them correctly classified fat as the most calorific nutrient [17]. Intervention studies have confirmed that improving nutrition education has a stronger impact on weight loss among obese populations [18]. Thus, dietetic consultations play a major role in improving nutrition knowledge. However, the effectiveness of these consultations has been seen to vary due to the practice being focused on diet planning and nutrition assessment, with minimal time given to nutrition education [19]. Therefore, it is essential to develop approaches that incorporate regular dietetic consultations, offering clients valuable opportunities to address their current challenges and goals and enhance their understanding of how nutrition knowledge and practices influence their food choices and overall health. This highlights the importance of implementing innovative strategies, such as continuous monitoring and health coaching [20], to strengthen the nutrition education provided by dietitians. Clients can achieve higher nutrition knowledge and sustain lifestyle changes to support long-term weight loss outcomes by integrating these strategies into a personalized, patient-centered approach within dietetic consultations [21].

Online nutrition education has recently been introduced as an alternative to in-person education, showing positive weight loss outcomes for obese and overweight patients. A previous study proved that digital nutrition education significantly improves nutrition knowledge using the knowledge assessment questionnaire (KAQ). In that study, nutrition education was provided via a developed CD-ROM, and significant improvements in nutrition knowledge were seen in the total number of participants after the intervention (*p* < 0.05) [22]. A study undertaken in Peru on teachers showed that a telehealth intervention improved participants’ knowledge and BMI [23]. Nonetheless, general nutrition knowledge needs to be improved worldwide [24,25]. Previous studies have shown that online nutrition education is effective and affordable and produces positive weight loss outcomes [26]. Thus, the alignment of nutrition education and lifestyle health coaching is a new approach that has been shown to have a strong impact on weight loss, which is worth investigating. The literature has confirmed the beneficial use of engagement strategies for digital education, such as telemonitoring or reminders and health coaching [27]. Combining telemonitoring and health coaching has been previously tested in overweight employees, showing significant long-term weight loss [28]. A recent literature review confirmed that combining telemonitoring and health coaching significantly impacts sustainable weight loss outcomes [29].

The primary outcomes of our pilot randomized controlled trial (RCT) [30] suggested that combining telenutrition, telemonitoring, and health coaching offers a comprehensive, personalized approach that can significantly enhance weight loss outcomes in overweight and obese individuals. Based on the summarized findings, we worked on the secondary outcomes of the same study, where we predicted that continuous weekly monitoring and monthly telehealth coaching, coupled with the delivery of online nutrition education, would improve general nutrition knowledge and that, in the context of dietary restriction, improving the understanding of nutritional concepts and behaviors would further enhance the efficacy of the dietary intervention.

## 2. Materials and Methods

### 2.1. Study Population and Design

This study was a 6-month pilot two-arm randomized controlled trial (RCT) carried out in Jeddah, Saudi Arabia, between January 2022 and August 2023. The study protocol was approved by the Research Ethics Committee (REC) at the Unit of Biomedical Ethics, the Faculty of Medicine at King Abdulaziz University (HA-02-j-008). A detailed description of the study protocol has been published by the same research group in the British Journal of Nutrition [31]. The trial was conducted in the Food, Nutrition, and Lifestyle Unit at the King Fahd Medical Research Center, Jeddah, Saudi Arabia. The protocol inclusion criteria included obese or overweight participants based on the WHO body mass index (BMI) criterion (a BMI of 25 or more was considered overweight, and 30 or more was considered obese). We included adults aged 20 to 50 years old of both genders. Meanwhile, we excluded participants who were not familiar with using online applications, those who had a history of chronic diseases, such as diabetes or cardiovascular diseases, thyroid dysfunction, or any other endocrine abnormality, those who were pregnant and lactating, and those who joined weight loss programs or used medication for weight loss during the past 3 months. Participant recruitment took place via the official online platforms of King Abdulaziz University and the King Fahd Medical Research Center, Jeddah, Saudi Arabia. All recruited participants were invited to a screening visit for a medical assessment and full anthropometric measurements. The measurements included systolic and diastolic blood pressure, weight, waist circumference, BMI, and body fat percentage, detected with the body composition analyzer. The eligible participants were randomly divided into two groups (the intervention and control groups). Both groups were provided with a hypocaloric diet and 3 nutrition education sessions via telenutrition (remotely). The intervention group was supported with weekly telemonitoring (a total of 36 weeks of telemonitoring) and monthly telehealth coaching (a total of 6 sessions) by both registered dietitians (RDs) and integrative nutrition health coaches. Telemonitoring was conducted using WhatsApp via smartphones, where health measures, such as weight, weekly steps, and blood pressure, were collected weekly. Telehealth coaching sessions were conducted via the video conference Zoom platform. The sessions included guidance and support to tackle different lifestyle aspects while following the dietary plan, which indirectly enforced the awareness and general nutrition knowledge gained during this study. Once participants were eligible to join this study, they were invited to 3 visits during the 6-month trial period—at baseline, after 3 months, and after 6 months—to measure their general nutrition knowledge and anthropometric measurements.

The sample size calculations for this study were estimated based on the primary outcome, “weight loss”, and secondary outcome, “general nutrition knowledge”, of the main study following a published protocol in the British Journal of Nutrition [30,31,32]. This study included an integrative approach, where weekly telemonitoring and monthly telehealth coaching were designed to achieve significant changes in weight, lifestyle factors, and general nutrition knowledge. Firstly, the power calculations to achieve weight reductions of approximately 3.7 kg (SD = 2.5) required a minimum of 35 participants per group [28]. Secondly, the power calculations to achieve significant differences in GNKQ scores between the two groups, seen between high- and low-ranking coaches (*p* < 0.001), also required a minimum of 35 participants per group [33]. Both calculations were estimated based on 80% power, a 5% significance level, and a 25% dropout rate. While this study was part of a pilot randomized controlled trial (RCT), we aimed to examine 10–30% of the measured sample size based on published recommendations on pilot study sample size calculations [34]. The aim of conducting a pilot study was to evaluate a newly developed approach, the “Integrative Model”, within a selected population for the first time. To ensure a representative sample and account for potential attrition, 70 participants were recruited, and 50 participants were eligible to enroll, with 25 participants allocated to each group. This study aimed to assess a secondary outcome, “general nutrition knowledge”, in the same cohort of overweight and obese participants in the main pilot study to also assess the associations with anthropometric measures. The total retention rates varied across the 3- and 6-month follow-up points. A total of 30 participants (18 in the intervention group and 12 in the control group) had completed the assessment at the 3-month follow-up. The number of completers had decreased to 25 (16 in the intervention group and 9 in the control group) by the 6-month follow-up. Participants who completed the 3-month follow-up were not necessarily the same as those who completed the 6-month follow-up. However, there was overlap between the groups, as some participants completed the 3-month assessment but not the 6-month assessment, and few of those who completed the 6-month assessment did not complete the 3-month assessment.

### 2.2. Online Nutrition Education

A total of 3 online nutrition education sessions were delivered during the whole 6-month trial period by both registered clinical dietitians (RDs) and integrative nutrition health coaches via the Zoom platform. At the end of each time-point visit (the baseline, 3-month, and 6-month visits), the research assistants created a Zoom meeting link and shared it with all participants from both the intervention and control groups via WhatsApp to attend the session. We standardized the sessions by assigning the same educator and providing the same educational material to all participants in both groups, and we recorded all participants’ attendance in each session to ensure they all received the same nutrition education session. The nutrition education sessions were 45 min long, and an additional 15 min was provided for all participants for discussions. The sessions’ content was demonstrated in a PowerPoint presentation, detailed as follows.

Session 1: Healthy grocery shopping and food choices.

This session focused on concepts of healthy food choices, such as nutrient-dense foods and fresh and seasonal produce, while avoiding processed foods. Participants were guided on how to prepare a well-organized shopping list and a meal plan to support their dietary goals.

Session 2: Reading food labels.

This session focused on food label components, such as serving size, calorie count, and macronutrient breakdown, alongside identifying hidden sugars, unhealthy fats, and excessive sodium. Participants were guided on how to read food labels and interpret nutrition information to support their health goals.

Session 3: The relationship between diet and disease.

This session focused on achieving a balanced diet using different tools such as the “Food Pyramid”, “Eatwell Guide”, or “MyPlate”, alongside demonstrating the impact of a balanced diet on overall health and chronic disease prevention. Participants were guided on how to control portions and enhance health via simple dietary adjustments, such as reducing sodium intake or adding more whole foods to support both dietary and health goals.

### 2.3. The Arabic General Nutrition Knowledge Questionnaire (GNKQ)

General nutrition knowledge was measured using the published Arabic version of the revised General Nutrition Knowledge Questionnaire [12]. The method was previously evaluated in 2020 for its reliability and validity to be used specifically for adults and was found to be suitable for use in adults and different Middle Eastern Arab countries. The GNKQ contained four sections: (1) recommendations and portion sizes for the main food groups; (2) specific types of food and their salt, fat, protein, and sugar contents; (3) healthy food choices; and (4) food quality and associations with increasing/decreasing risk of chronic diseases and weight management. The questionnaire consisted of 88 items distributed among the four sections, with 18 questions in the first section, 36 in the second, 13 in the third, and 21 in the last. The questions had a multiple-choice design, requiring only one answer. Given that the GNKQ is a self-reported questionnaire, a research assistant was assigned to provide clear instructions and sufficient time for all participants to complete the questionnaire during each visit (after baseline, after 3 months, and after 6 months) to reduce subjectivity due to misinterpretation or fast responses. Thus, the GNKQ was filled in at the Food, Nutrition, and Lifestyle Unit at the King Fahd Medical Research Center, Jeddah, Saudi Arabia.

### 2.4. Statistical Analysis

The data were analyzed using the SPSS program version 26.0. Continuous data were reported as means and SD. Between-group differences in baseline continuous characteristics were examined using an independent *t*-test. Baseline categorical variables were reported as frequencies and percentages (%) and examined using the chi-square test. The general nutrition knowledge (GNK) scores were presented as means and SD. A repeated-measures Friedman test was conducted on all time-point completers as the primary analysis. The within-subjects factor was time (baseline, 3 months, and 6 months), with pairwise comparisons between the time-points. The between-subjects factor was the intervention group (the intervention and control groups). The *p*-values were adjusted for pairwise comparisons using Bonferroni correction. Secondary analysis was conducted on completers at any time-point to maximize the data utilization. The Wilcoxon signed-rank test was used to assess differences in GNKQ scores between baseline and subsequent time-points (3 and 6 months) within each group due to the data’s non-normal distribution. The Mann–Whitney U test was utilized to compare the median change in knowledge scores between the intervention and control groups at both 3 and 6 months. A series of regression analyses were conducted to investigate the relationship between changes in nutrition knowledge scores and various anthropometric measurements. The dependent variables included ranked changes in weight, BMI, fat percentage, muscle percentage, visceral fat percentage, and waist circumference after 3 months of intervention. The independent variable was the ranked change in the nutrition knowledge score after 3 months of intervention. A *p*-value of less than 0.05 was considered statistically significant.

## 3. Results

### 3.1. Characteristics of Participants at Baseline

A total of 30 participants (18 and 12 in the intervention and control arms, respectively) completed the 3-month visit, and 25 (16 and 9 in the intervention and control arms, respectively) completed the 6-month visit. Both arms were balanced, as the participants’ baseline characteristics did not significantly differ between the two study groups, as shown in Table 1.

### 3.2. Weight, BMI, and WC at All Time-Points for All Time-Point Completers

While this manuscript’s primary focus was the general nutrition knowledge outcomes, Table 2 provides a summary of the weight-related outcomes. Briefly, participants in the intervention group only showed significant reductions in weight, BMI, WC, and fat% and a significant increase in muscle% at 3 months from baseline but not at 6 months. The effect of the intervention on weight loss and anthropometric measurements was reported in the main pilot study [30,32].

### 3.3. General Nutrition Knowledge Scores

#### 3.3.1. GNKQ Scores at All Time-Points for All Time-Point Completers

Overall, the GNKQ results showed modest improvements in both the intervention and control groups over time, with no significant between-group differences in the change from baseline at 3 or 6 months. Scores in the intervention group generally increased from baseline to 3 and 6 months, including a significant overall effect of time on both overall nutrition knowledge and the diet–disease relationship and weight management sections (*p* < 0.05; Table 3). However, the within-group comparisons did not reach statistical significance. The GNKQ scores in the control group did not significantly change at either the 3- or 6-month time-points (Table 3).

#### 3.3.2. GNKQ Scores for All Time-Points for Any Time-Point Completers

Table 4 describes the changes in GNKQ scores from baseline to 3 and 6 months across different sections for the intervention and control groups and all participants combined who completed assessments at any time-point (n = 30 at 3 months; n = 25 at 6 months). Significant improvements in GNKQ scores were observed in certain areas and time-points across the study groups. At 3 months, significant increases were noted in the combined groups for dietary recommendations (*p* = 0.035), food groups (*p* = 0.027), the diet–disease relationship and weight management (*p* = 0.014), and overall nutrition knowledge (*p* = 0.003). Additionally, the intervention group showed a significant improvement in the diet–disease relationship and weight management nutrition knowledge at this time-point (*p* = 0.007), while the control group demonstrated a significant increase in food groups (*p* = 0.028) and overall nutrition knowledge (*p* = 0.019). At 6 months, significant improvements in nutrition knowledge were limited to the diet–disease relationship and weight management section for the intervention group (*p* = 0.026), with a near-significant trend in the combined groups (*p* = 0.055) (Table 4).

The change in knowledge scores between the pre-intervention and post-intervention periods was analyzed using the Mann–Whitney U test to compare the effectiveness of the intervention and control groups. There was no significant difference in the change in knowledge scores between the intervention and control groups at both 3 and 6 months.

### 3.4. Relationship Between Changes in Nutrition Knowledge Scores and Various Anthropometric Measurements

This analysis aimed to identify the relationship between changes in nutrition knowledge scores and various anthropometric measurements. There was no significant association between the change in nutrition knowledge scores and changes in weight, muscle percentage, or waist circumference after 3 months of intervention. However, a significant negative association was observed between changes in nutrition knowledge scores and both BMI (β = −0.746, SE = 0.320, 95% CI [−1.405, −0.087], r = 0.415, and *p* = 0.028; Table 5) and visceral fat percentage (β = −0.839, SE = 0.245, 95% CI [−1.344, −0.333], r = 0.573, and *p* = 0.002; Table 5). Additionally, there was a marginally significant trend indicating that increased nutrition knowledge might be associated with a reduced fat percentage (β = −0.620, SE = 0.331, 95% CI [−1.301, 0.061], r = 0.345, and *p* = 0.073; Table 5 [SE1]).

## 4. Discussion

This study aimed to examine general nutrition knowledge using a validated Arabic General Nutrition Knowledge Questionnaire GNKQ [12] in all participants in a pilot randomized controlled trial (RCT). The results show modest improvements over time in both the intervention and control groups, with no significant differences between the groups at 3 or 6 months. While within-group differences approached significance at 3 months compared with baseline (*p* = 0.055 and 0.098, respectively), they showed no significant changes at 6 months. Conversely, the GNKQ scores in the control group remained relatively unchanged, with no significant improvements observed at either the 3- or 6-month visits. Consistent with our study, a cross-sectional study conducted on nursing students living in the UAE indicated an overall nutrition knowledge score of 53.86 (19.44), which is very similar to the overall nutrition knowledge of our participants after receiving the nutrition education sessions [35]. Hence, our participants successfully gained a similar level of nutrition-related information to students specialized in healthcare. Our findings were also consistent with a study carried out on students living in both the UAE University and Hashemite University in Jordan. Students with a nutrition background had higher GNKQ scores (66.0 (10.6)), whereas those without a nutrition background had low scores (38.0 (10.7)) (*p* < 0.001; d = 2.6) [12]. To compare this study’s participants with university students living in the UK, the overall nutrition knowledge score among UK students was found to be 64.0, similar to the previous study, confirming that a nutrition background significantly improves general nutrition knowledge [24]. Nonetheless, the characteristics of populations differ [36], and university students with a nutrition background are expected to have a higher nutrition knowledge level compared with Saudi participants. Several factors may have influenced the variations in participant characteristics, including age, gender, and nutrition literacy, which this study did not consider [37]. The GNKQ has been investigated among young men without tertiary education, where they obtained the lowest GNKQ scores [38]. Nutrition knowledge has also been examined via different tools among the Saudi population, and there is still a lack of knowledge and awareness regarding the correct identification of portion size and MyPlate guidelines [8]. Nutrition education is still not implemented in Saudi schools [6] due to a lack of awareness and the required training [7]. Thus, it is essential to highlight the importance of nutrition education and its impact on consumers’ behavior and healthy eating patterns. This study proved that education sessions focused on food choices, food labels, and healthy eating nutrition education sessions increase general nutrition knowledge scores. However, the lack of significant differences suggests that the improvements may not have been sustained, possibly due to diminishing engagement with the intervention or other external factors.

In this study, significant improvements were seen in different sections of the questionnaire. At the 3-month follow-up, participants in the control group still showed significant improvements in “overall nutrition knowledge” and “food groups knowledge” compared with their scores before joining the program. This may have happened due to the online nutrition education sessions provided for both groups. Meanwhile, participants in the intervention group who were supported with weekly telemonitoring and monthly telehealth coaching showed significant improvements in “diet–disease and weight management knowledge” at the 3- and 6-month follow-ups. All participants in both the intervention and control groups received the same three online nutrition education sessions, which explains the improvements in overall nutrition knowledge in both groups as well as the increase in overall nutrition and food group knowledge among the control group participants. Participants who only received their nutrition information from these education sessions might have mostly relied on them as a primary source of health-related information and, therefore, might have been more committed to comprehending and utilizing the information learned. Furthermore, participants in the intervention group with access to personalized health coaching had an additional channel for acquiring health-related information, which might explain the increase seen in the diet–disease relationship and weight management knowledge among these participants throughout the 6-month period. Conversely, a study undertaken among Chinese university students showed 60% correct answers for the GNKQ, but improvements were still needed regarding the relationship between diet and disease [39]. Previous work has shown that health coaching can contribute to improving chronic diseases, including reducing cardiovascular disease risk and hemoglobin A1c levels and normalizing blood pressure [40,41,42]. Our study was the first to establish the effect of health coaching alongside telenutrition and telemonitoring on increasing the general nutrition knowledge among individuals with overweight/obesity over six months. This finding aligns with previous reports that health coaching improved diabetes knowledge among individuals with diabetes [43]. Thus, online nutrition education may be an effective tool to enhance the overall nutrition knowledge among a population while also being accessible and convenient [44]. A randomized controlled trial among students mentioned that using a game-based e-program in nutrition knowledge was interesting and easy to understand [45]. Digital education programs can also reach populations with low incomes and living in distanced areas [46]. This can be provided via telenutrition, where a clear shift in healthcare services toward adopting telehealth has been seen globally [47]. In the Arab world, a cross-sectional study revealed that dietitians are now adopting alternative telenutrition approaches via social/mass media [48].

A significant correlation between nutrition knowledge scores and various anthropometric measurements was revealed at the 3-month visit, such as improvements in BMI and visceral fat percentage. Despite this, nutrition knowledge has been proven to impact food choices and eating behaviors. A study confirmed that dancers with eating disorders have lower nutrition knowledge, which impacts body weight and BMI [49]. Greater reductions in BMI and visceral fat might have been driven by behavioral changes rather than increases in knowledge due to the integration of both telemonitoring and health coaching in the intervention group. The intervention group showed a trend toward significant improvement in only overall nutrition knowledge while experiencing significantly greater reductions in weight, BMI, and fat percentage. These findings suggest that while improvements in nutrition knowledge may not significantly influence weight, muscle percentage, or waist circumference, they are likely to contribute to reductions in BMI and visceral fat percentage, with a potential effect on overall fat percentage. This suggests that the telemonitoring intervention may have directly influenced behavioral changes (e.g., improved diet or physical activity), leading to weight loss without necessarily requiring a significant boost in overall nutrition knowledge. In this case, telemonitoring might have encouraged behavioral compliance via regular feedback, monitoring, and personalized support, which could be more effective in short-term weight management than pure knowledge gains. Meanwhile, the control group showed significant improvements in nutrition knowledge but did not experience as much weight reduction. This may be because increased knowledge might not have translated into practical, sustained behavioral changes as effectively as telemonitoring. A study protocol was recently published on coaching and/or education intervention for obese parents with their children, but the research is still ongoing [50]. Like our study design and findings, the Smarter Pregnancy mHealth coaching program has been seen to improve women’s lifestyle and nutrition education via mobile health coaching [51]. In addition, the Healthy Supermarket Coach program has shown that health coaching and nutrition peer education in supermarkets improve nutrition knowledge and attitudes toward healthy eating patterns [52]. Health coaching evidently impacts lifestyle and behavioral changes, but little research has been conducted to assess nutrition knowledge.

Our study is considered the first to have explored how continuous guidance via weekly telemonitoring and monthly telehealth coaching supports weight loss while also enhancing general nutrition knowledge. This confirms that nutrition education alone is insufficient to drive behavioral changes, especially if it lacks direct and continuous support, which can be offered via telemonitoring and telehealth coaching, as seen among the intervention group. However, this study’s main limitation was the small sample size, considering that it was a pilot study, which examined 10–30% of the targeted sample size. It focused on assessing the effect of the intervention on weight measurements, with nutrition knowledge as a secondary outcome. Hence, the sample sizes were limited, particularly at the 6-month time-point, where fewer participants completed the assessment. The sample size discrepancy between 3 months (larger) and 6 months (smaller) could have influenced the consistency of significant findings across the time-points, as the significant improvements observed at 3 months were not consistently replicated at 6 months. This variation highlights that this study may have been underpowered to detect consistent changes in nutrition knowledge over time, particularly as a secondary outcome. Given these limitations, further studies with larger sample sizes are recommended to validate these preliminary findings on nutrition knowledge. Future research should also examine whether these changes are sustained beyond the short term and whether they are truly attributable to the intervention rather than sample size fluctuations. Regression analyses were conducted only for the 3-month time-point, as initial within- and between-group analyses at 6 months did not show significant intervention effects on the GNKQ scores or anthropometric measurements. Consequently, we did not conduct regression analysis for the 6-month mark, as it would likely not have yielded meaningful findings. These results emphasize that further studies with larger sample sizes and longer follow-up periods are needed to explore potential long-term relationships between changes in nutrition knowledge and anthropometric measurements. The GNKQ is a commonly and widely used instrument to assess general nutrition knowledge but is still considered subjective due to being based on self-reporting. This suggests that assessment tools should be developed and validated for nutrition knowledge before and after attending online nutrition education sessions.

## 5. Conclusions

The current pilot RCT examined the impact of weekly telemonitoring and monthly telehealth coaching on improving general nutrition knowledge using an Arabic-validated GNKQ. The findings demonstrate improvements in general nutrition knowledge over time among overweight and obese participants, particularly in the intervention group. Furthermore, significant associations between the GNKQ and anthropometric measurements were revealed, highlighting the importance of enhancing nutrition knowledge as part of weight loss strategies. Future intervention studies must conduct qualitative analysis to further understand the impact of telemonitoring and health coaching on improving knowledge and translating acquired health and nutrition information into action and behavioral changes from the participants’ perspective.

## Figures and Tables

**Table 1:** Baseline characteristics of all participants (GNKQ completers).

	3-Month Completers				6-Month Completers			
	Intervention n = 18 (Mean ± SD)		Control n = 12 (Mean ± SD)		Intervention n = 16 (Mean ± SD)		Control n = 9 (Mean ± SD)	
	Male n = 6	Female n = 12	Male n = 7	Female n = 5	Male n = 5	Female n = 11	Male n = 6	Female n = 3
**Age** (years)	27 (6)	34 (13)	37 (8)	33 (11)	29 (6)	36 (13)	39 (9)	40 (9)
**Weight** (kg)	107 (26.8)	84.3 (14.4)	99.9 (26.2)	80.2 (8.8)	107 (27)	82.8 (15.3)	102 (24)	75.3 (4.1)
**BMI** (kg/m^2^)	34.3 (6.99)	33.3 (5.9)	34.2 (7.1)	33.6 (3.9)	34.3 (7)	32.5 (6.6)	34.2 (7)	31.9 (3.7)
**Fat%**	37 (7)	48.9 (5.6)	36.7 (7.4)	49.2 (5.7)	37 (7)	47.6 (6.5)	36.8 (7.4)	47.7 (6.5)
**Muscle%**	30 (3.7)	22 (2.3)	29.2 (3.4)	22 (2.8)	30 (4)	22.6 (2.7)	29 (3.1)	22.4 (3.4)
**Visceral fat%**	15.4 (4.6)	9 (4)	17 (6)	9 (2)	15 (5)	9 (4)	17 (6)	9 (3)
**WC** (CM)	112.1 (18.8)	90.7 (11.7)	113.7 (16.8)	90 (6.3)	112.1 (19)	89.8 (12)	114 (16.4)	88 (7.8)
**Sys BP**	126 (12.7)	135 (18)	132 (16)	133 (19)	126 (13)	130 (17)	130 (17)	135 (26)
**Dias BP**	80 (11.9)	78 (15)	86 (5)	92 (14)	80 (12)	75 (14)	84 (6)	92 (18)

Data are presented as means ± SD. Statistical comparisons between the intervention and control groups were conducted using independent samples *t*-tests (for normally distributed variables) or Mann–Whitney U tests (for non-normally distributed variables) within each sex category at baseline. No statistically significant differences were observed between the intervention and control groups for any baseline characteristic (all *p* > 0.05).

**Table 2:** Mean, SD, and statistical significance values for within- and between-group differences in weight, BMI, and WC at all time-points for all time-point completers.

	Baseline Mean (SD)	3 Months Mean (SD)	Within-Group Analysis Baseline vs. 3 Months *p*-Value	6 Months Mean (SD)	Within-Group Analysis Baseline vs. 6 Months *p*-Value	Between-Group Analysis Intervention vs. Control
**Weight**						
Intervention	91.5 (22.3)	87.3 (20.7)	0.015 *	88.5 (22.9)	0.227	0.620
Control	94.7 (23.9)	94.2 (25)	1	93.4 (24.5)	0.696	
**BMI**						
Intervention	33.6 (6.5)	32 (6.6)	0.012 *	32.4 (7.5)	0.253	0.624
Control	34.4 (5.7)	34.1 (6)	1	33.8 (5.7)	0.528	
**WC**						
Intervention	97 (18)	92 (16)	0.002 **	93 (16)	0.112	0.23
Control	106 (20)	103 (19)	0.081	101 (20)	0.217	
**Fat%**						
Intervention	44.7 (8.3)	41.6 (8.8)	0.004 **	43 (9.7)	0.199	0.634
Control	41.9 (8)	40.8 (8.2)	0.27	41.2 (7.9)	1	
**Muscle%**						
Intervention	25 (5)	26.9 (5.3)	0.016 *	25.5 (6.5)	1	0.805
Control	26 (4.1)	26.7 (4.6)	0.212	26.5 (4.2)	0.662	
**Visceral fat%**						
Intervention	11.4 (4.7)	10.3 (4.2)	0.071	10.5 (4.5)	0.181	0.11
Control	14.8 (6.2)	14.8 (6.4)	1	14.4 (6.3)	0.239	

The data are expressed as means (SD) for the DASS-21 scores of the completers of this study (n = 15) in the intervention and (n = 8) control groups. *p*-values were obtained via repeated-measures analysis of variance (ANOVA) for within-group comparisons with Bonferroni correction for multiple comparisons. Between-group comparisons were conducted using independent *t*-tests. (*p*-values: * *p* < 0.05 and ** *p* < 0.01).

**Table 3:** Mean, SD, and statistical significance values for within- and between-group differences in GNKQ scores within each study group (intervention and control groups) as well as for all participants combined at all time-points for all time-point completers.

GNKQ Section	Baseline	3 Months	Within-Group Analysis Baseline vs. 3 Months *p*-Value	6 Months	Within-Group Analysis Baseline vs. 6 Months *p*-Value	Overall Time Effect	Difference at 3 Months from Baseline	Between-Group *p*-Value Intervention vs. Control at 3 Months	Difference at 6 Months from Baseline	Between-Group *p*-Value Intervention vs. Control at 6 Months
**Dietary recommendations**										
Intervention	8.73 (2.89)	9.33 (2.72)	NP	9.4 (2.75)	NP	0.133	0.6 (2.1)	0.506	0.67 (4.03)	0.728
Control	9.63 (3.29)	10.87 (2.17)	NP	10.5 (1.41)	NP	0.497	1.25 (2.82)		0.88 (2.9)	
Combined groups	9.04 (2.99)	9.87 (2.6)	NP	9.78 (2.39)	NP	0.12	0.83 (2.33)		0.74 (3.61)	
**Food groups**										
Intervention	21.5 (6.8)	21.9 (7.5)	NP	21.9 (8.2)	NP	0.942	0.33 (2.06)	0.265	0.33 (3.7)	0.591
Control	21.5 (4.4)	23.3 (2.7)	NP	22.3 (3.2)	NP	0.629	1.75 (3.11)		0.75 (4.71)	
Combined groups	21.5 (5.99)	22.4 (6.2)	NP	22 (8.8)	NP	0.846	0.83 (2.5)		0.48 (3.98)	
**Healthy food choices**										
Intervention	5.67 (2.09)	5.8 (1.97)	NP	6.13 (2.17)	NP	0.365	0.13 (1.64)	0.728	0.47 (1.85)	0.428
Control	5.75 (0.89)	5.5 (1.31)	NP	5.75 (1.28)	NP	0.961	−0.25 (1.04)		0 (1.31)	
Combined groups	5.7 (1.74)	5.7 (1.74)	NP	6 (1.88)	NP	0.5	0 (1.45)		0.3 (1.66)	
**Diet–disease relationship and weight management**										
Intervention	13.7 (4.8)	15.5 (3.4)	0.107	15.3 (4.5)	0.134	0.034 *	1.8 (2.18)	0.265	1.53 (2.39)	0.169
Control	13.9 (1.5)	14.4 (2.6)	NP	13.4 (4.2)	NP	0.725	0.5 (2.78)		−0.5 (3.51)	
Combined groups	13.8 (1.46)	15.1 (3.11)	0.098 ^a^	14.6 (4.4)	0.196	0.042 *	1.35 (2.42)		0.83 (2.92)	
**Overall nutrition knowledge**										
Intervention	51.2 (15.8)	53.9 (14.4)	0.053 ^a^	54.2 (15.7)	0.134	0.034 *	2.67 (5.42)	0.975	3 (8.72)	0.466
Control	51.9 (8.4)	55.6 (5.8)	NP	53.3 (8)	NP	0.177	3.75 (6.34)		1.38 (6.91)	
Combined groups	51.4 (13.5)	54.5 (5.8)	0.055 ^a^	53.9 (13.3)	0.314	0.048 *	3.04 (5.64)		2.43 (8.01)	

The data are expressed as means (SD) for the GNKQ scores of the completers of this study (n = 15) in the intervention and (n = 8) control groups. The *p*-values were obtained using the Friedman test for within-group analyses, with Bonferroni correction for pairwise comparisons between time-points. NP indicates that multiple comparisons were not performed due to a non-significant overall time effect. Significant *p*-values are in bold (*p*-values: ^a^ *p* > 0.05 and * *p* < 0.05).

**Table 4:** Changes in GNKQ scores from baseline to 3 and 6 months across different sections for the intervention and control groups and all participants combined who completed assessments at any time-point (n varied by time-point).

GNKQ Section	Time-Point Comparison	Group	n	GNKQ Score		*p*-Value for Change
				Baseline GNKQ Score Mean (SD)	Post-Time-Point GNKQ Score Mean (SD)	
Dietary recommendations	3-month comparison	Intervention	18	8.5 (2.8)	9.4 (2.6)	0.131
		Control	12	9.8 (2.9)	10.9 (2)	0.124
		Combined groups	30	9 (2.8)	10 (2.5)	0.035 *
	6-month comparison	Intervention	16	9 (3)	9.4 (2.7)	0.173
		Control	9	9.2 (3.3)	10.2 (1.6)	0.339
		Combined groups	25	9.1 (3)	9.7 (2.3)	0.103
Food groups	3-month comparison	Intervention	18	20.3 (7.5)	21.2 (7.2)	0.31
		Control	12	21.8 (3.6)	23.7 (2.3)	0.028 *
		Combined groups	30	20.9 (6.2)	22.2 (5.8)	0.027 *
	6-month comparison	Intervention	16	21.6 (6.6)	21.8 (7.9)	0.68
		Control	9	19.9 (6.4)	21.3 (4)	0.674
		Combined groups	25	21 (6.4)	21.6 (6.7)	0.59
Healthy food choices	3-month comparison	Intervention	18	5.5 (2)	5.8 (1.9)	0.47
		Control	12	5.7 (1.3)	6.2 (1.7)	0.286
		Combined groups	30	5.6 (1.7)	6 (1.8)	0.208
	6-month comparison	Intervention	16	5.6 (2)	6.1 (2.1)	0.161
		Control	9	5.1 (2.1)	5.2 (2)	0.792
		Combined groups	25	5.4 (2)	5.8 (2.1)	0.18
Diet–disease relationship and weight management	3-month comparison	Intervention	18	13.2 (4.6)	14.8 (3.5)	0.007 **
		Control	12	14.6 (2.4)	14.9 (2.6)	0.558
		Combined groups	30	13.8 (3.8)	14.9 (3.1)	0.014 *
	6-month comparison	Intervention	16	13.8 (4.7)	15.3 (4.3)	0.026 *
		Control	9	13.1 (2.7)	13.3 (3.9)	0.723
		Combined groups	25	13.6 (4)	14.6 (4.2)	0.055
Overall nutrition knowledge	3-month comparison	Intervention	18	48.8 (16)	52.4 (13.5)	**0.055 ^a^**
		Control	12	53 (7.6)	57.5 (6.1)	0.019 *
		Combined groups	30	50.5 (13.3)	54.5 (11.3)	0.003 **
	6-month comparison	Intervention	16	51.6 (15.4)	54.2 (15.1)	0.103
		Control	9	48.3 (13.2)	51.3 (9.5)	0.475
		Combined groups	25	50.4 (14.4)	53.2 (13.2)	0.071

The data are expressed as means (SD) for the GNKQ scores. The table includes data from all participants who completed assessments at any time-point (baseline, 3 months, or 6 months). In contrast, Table 3 reports data from participants who completed assessments at all three time-points. The significant differences between baseline and subsequent time-points (3 and 6 months) in the GNKQ score within each group were obtained using the Wilcoxon paired test and are shown in bold (*p*-values: ^a^ *p* > 0.05, * *p* < 0.05, and ** *p* < 0.01).

**Table 5:** Regression analyses examining the relationship between changes in GNKQ scores and anthropometric measurements after 3 months of intervention.

Variable	β	SE	95% CI	r	*p*-Value
Change in weight	−0.263	0.19	[−0.653, 0.127]	0.263	0.177
Change in BMI	−0.746	0.32	[−1.405, −0.087]	0.415	0.028 *
Change in fat %	−0.62	0.331	[−1.301, 0.061]	0.345	0.073 ^a^
Change in muscle %	0.509	0.316	[−0.143, 1.160]	0.312	0.12
Change in visceral fat	−0.839	0.245	[−1.344, −0.333]	0.573	0.002 **
Change in WC	−0.476	0.336	[−1.166, 0.215]	0.268	0.169

The dependent variables include ranked changes in weight, BMI, fat percentage, muscle percentage, visceral fat percentage, and waist circumference. The independent variable is the ranked change in the overall nutrition knowledge score. The regression coefficient (β), standard error (SE), 95% confidence interval (CI), correlation coefficient (r), and *p*-values are reported (* *p* < 0.05 and ** *p* < 0.01).

## Data Availability

The data presented in this study are available upon reasonable request from the corresponding author (ooaeid2@kau.edu.sa).
